# Asthma is associated with a lower incidence of metastatic colorectal cancer in a US patient cohort

**DOI:** 10.3389/fonc.2023.1253660

**Published:** 2023-10-04

**Authors:** Jacob Beckstead, Kunaal Mehrotra, Kayla Wilson, Barbara Fingleton

**Affiliations:** Program in Cancer Biology, Department of Pharmacology, Vanderbilt University, Nashville, TN, United States

**Keywords:** adenocarcinoma, asthma, cancer, colorectal, electronic medical record, metastasis

## Abstract

In previous pre-clinical studies, we examined the contribution of interleukin 4 receptor (IL4R) signaling in the progression and metastasis of colorectal cancer (CRC). Aberrant activation of this receptor can result in atopic diseases such as asthma. We hypothesized that further evidence for the contribution of excessive IL4R being associated with CRC progression could be seen in medical records, and specifically that chronic asthma patients were more likely to be diagnosed with metastatic CRC. To test this hypothesis, we took advantage of the Synthetic Derivative, a resource developed at Vanderbilt University Medical Center that hosts de-identified data taken from the electronic medical record. We developed search protocols that produced retrospective cohorts of invasive CRC patients and cancer-free equivalents. In comparing 787 metastatic CRC patients to 238 non-metastatic patients, we actually found significantly fewer asthmatics went on to develop metastatic CRC (P=0.0381). By comparing these groups together against 1197 cancer-free patients, even fewer asthmatic patients would develop invasive CRC (P<0.0001). While these results are clearly in opposition to our original hypothesis, they still support a link between chronic asthma and metastatic CRC development. One intriguing possibility, that will be examined in the future, is whether treatment for chronic asthma may be responsible for the reduction in metastatic cancer.

## 1 Introduction

Colorectal cancer (CRC) remains the second deadliest cancer worldwide (1). Survivorship has greatly increased thanks to improved treatments and screening protocols, but patient outcome remains heavily influenced by diagnostic stage. The 5-year survival rate for patients in the United States diagnosed when CRC has invaded the regional lymph nodes or distant tissues plummets from 91% to 72% and 14%, respectively. Approximately 60% of patients are initially diagnosed at these later two stages. Recent advances have honed in on the physiological and biochemical pathways exploited by CRC to facilitate metastatic spread (2–5) and we have previously suggested that interleukin (IL)-4 receptor (IL4R) signaling is one such pathway (6–8).

Activation of the IL-4 signaling pathway in B cells is known to increase IgE, whose overproduction has been a longstanding hallmark of atopic diseases such as asthma (9–11). It is estimated that 14.2% of adults in the United States have been diagnosed with asthma in their lifetime, with 8.4% holding a current diagnosis and 40.7% of those experiencing at least one asthma attack a year (12). Despite the prevalence of asthma, the literature is unclear on potential influences on cancer risk, and of the studies done, the majority have focused on lung cancer (13–15). Interestingly, increased IgE has been linked both with increased and decreased cancer risk, with several hypotheses proposed to explain these opposing findings, including increased inflammation and Th2 skewing as pro-cancer events, and increased immune surveillance and removal of potential carcinogens by coughing/sneezing as anti-cancer events (16). Recently reported studies have demonstrated a positive correlation between asthma and future cancer diagnosis including in colon cancer, but do not account for disease progression or patient outcome (17–19). Additionally, studies of polymorphisms in *IL4R* have suggested that at least some are associated both with increased prevalence of asthma (20, 21) and in separate cohort studies, with increased risk of colon cancer (22, 23). Based on our previous *in vitro* and animal studies suggesting a pro-metastatic role for IL4R signaling, we hypothesized that the enhanced IL-4 pathway activity in asthmatic patients would put them at greater risk of developing colorectal metastasis if they were diagnosed with early-stage colon cancer. Notably, this hypothesis can be expanded to also include other ramifications of asthma and allergy such as increased IgE levels, but the central question is whether colon cancer progression to metastasis is promoted in asthmatic patients.

Here we describe our use of Vanderbilt University Medical Center(VUMC)’s Synthetic Derivative (SD) to investigate this question. The SD is a research resource containing the de-identified clinical information from Vanderbilt’s electronic medical record, currently hosting over 3.5 million individuals. Our search revealed an apparent correlation between asthma and the development of metastatic disease in colorectal cancer patients. Unexpectedly, however, the correlation was a negative one and raises new questions for the future.

## 2 Materials and methods

**Figure 1:**
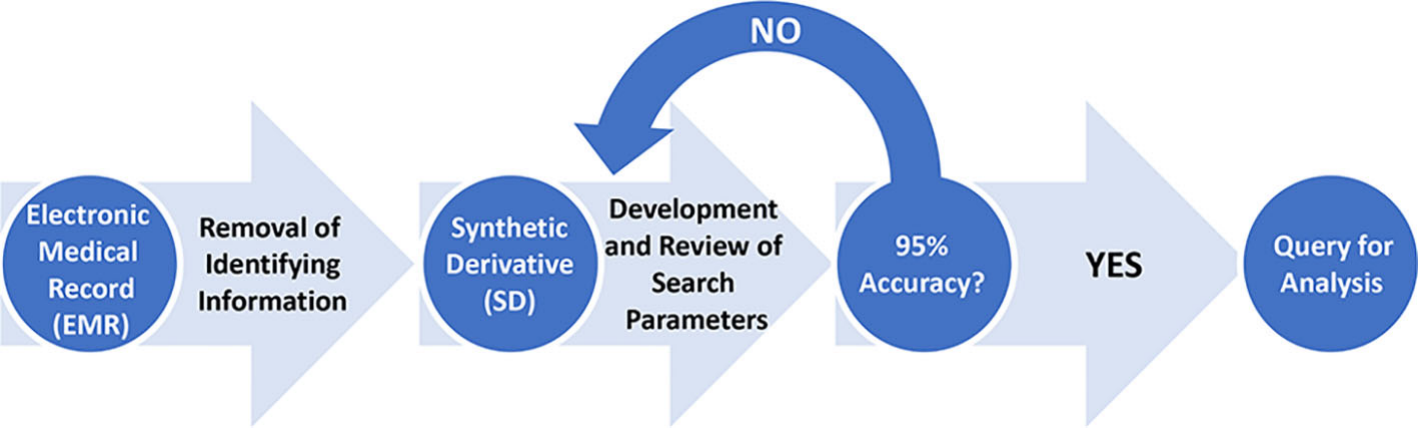
Methodology schematic of search protocol development. Once initial database search parameters are established, the resulting records are manually reviewed to confirm the accuracy of queried records to the desired phenotype. The protocol continues to be refined until over 95% of records pulled from the SD match the desired phenotype. These records are then saved for full analysis.

We developed two search protocols to select patients with colorectal adenocarcinoma between 2005 and 2015, dependent on whether their disease progressed to metastasize lymph nodes or regional or distant tissues. Final patient data was queried from SD Discover on August 18th, 2021 using the search parameters provided below and in the supplement. The International Classification of Disease (ICD) codes have long served as the standard of disease categorization in medical research. However, errors are known to populate the record, particularly in the transition from paper records to electronic databases. As such, we required that two or more incidents of the desired codes be present in all of our search protocols and lab members manually reviewed these patients’ de-identified records to ensure a greater than 95% accurate selection of patients for replicable study (**Figure 1**).

To ensure that patients who developed only early-stage CRC did not skew the results and that we only pulled SD records of patients with invasive CRC, records had to meet the following criteria for each of the cohorts: Records included in the metastatic cohort must contain two or more CRC ICD codes (**Supplementary Table 1**) between January 1^st^, 2005 and December 31^st^, 2015 and two or more ICD codes for cancer metastasis or related keywords. (**Supplementary Table 2**) Included records must also include the keyword ‘colorectal’ in related pathology reports to reduce the confounding effect of multiple types of cancer diagnoses. Records included in the non-metastatic invasive CRC cohort similarly required two or more CRC ICD codes within the studied time frame, (**Supplementary Table 1**) but with the exclusion of ICD codes and keywords related to metastasis and underdeveloped polyps and tumors. (**Supplementary Table 3**) These records must also contain keywords related to tumor grading (‘pt2’, ‘pt3’, and ‘pt4’) in outpatient and inpatient notes, pathology notes, and notes general to the medical record as well as the keyword ‘n0’ in the same documents to ensure we gathered records that had sufficiently developed tumors but had not metastasized to regional lymph nodes.

Our search parameters allowed for combining these two CRC groups together to form a single invasive CRC group to compare to a cancer-free control. An ICD 10 code unrelated to cancer (S00.00X - Unspecified superficial injury of scalp) was used to pull a random selection of cancer-free patients from SD Discover records April 4th, 2020, excluding any records with ICD 10 neoplasm codes (C00-D49) or associated CMS GEM Crosswalk ICD 9 codes. These records also underwent independent manual review by lab members to confirm cancer-free status.

Patients in our CRC cohorts were designated as asthmatic if they received at least two relevant ICD codes (ICD 9 493 and/or ICD 10 J44 and J45) at least one year prior to CRC diagnosis. We calculated the average age patients received their initial CRC diagnosis to set an upper age threshold for cancer-free patients’ asthma diagnoses, ensuring accurate comparison to the number of patients diagnosed with asthma prior to CRC development. Cancer-free patients diagnosed with asthma after this age threshold were not considered asthmatic for the purpose of this study. To mitigate the effects of immortal time bias, cancer-free patients were only included whose age exceeded this value at the start of our study and were unlikely to develop CRC after our study concluded. Preliminary analysis was also performed while stratifying for sex and race to ensure our cohorts were an accurate representative of the Synthetic Derivative and to identify any groups at particular risk.

Statistical analysis was performed using GraphPad Prism version 9.2.0 (GraphPad Software, San Diego, California USA, www.graphpad.com). Differences between groups were compared using Fisher’s exact test.

## 3 Results

For this work, we utilized a research resource containing the de-identified clinical information from Vanderbilt’s electronic medical record, currently hosting over 3.5 million individuals (24). Having removed identifying information for the patient and practitioners, the electronic medical record as provided by the SD contains primary documentation of a patient’s disease, including pathology reports and applied treatments. The ICD published by the World Health Organization provides standardized codes for diagnostic reporting used in medical research. We first identified ICD codes that could identify patients that were diagnosed with colon, colorectal or rectal cancer. Using additional notes provided by the SD Discover program, we could not only select for these diagnostic ICD codes, but also categorize by disease progression. In this way, we were able to eliminate confounding patients whose disease was detected and removed early and so did not progress beyond *in situ* polyp formation. It is important to note that all records returned after each query of the SD were individually assessed to ensure that they met the criteria of colorectal cancer that was beyond a stage I polyp. After delineating cohorts of patients with at least stage II colorectal cancer that did or did not become metastatic, we then identified ICD codes signifying a diagnosis of asthma. To ensure that single acute episodes were not included, we only designated a patient as having asthma if there were at least two consecutive clinic visits using a relevant ICD code at least one year prior to their first CRC ICD code.

Overall, our review resulted in 787 patients with metastatic CRC and 238 patients whose disease progressed but did not become metastatic. 6 patients in each cohort received more than one asthma diagnosis at least one year prior to their CRC diagnosis. Contrary to our expectations, significantly fewer metastatic patients had received asthma diagnoses prior to CRC. (P=0.0381, **Table 1**).

**Table 1:** Asthma-free *vs*. Asthmatic patients in our colorectal cancer (CRC) and Cancer-free search cohorts.

Asthma-Free: Asthmatic (%)	Metastatic CRC	Non-Metastatic CRC	Cancer-free	P: Metastatic risk	P: CRC risk
Total	781: 6 (0.76%)	232: 6 (2.52%)	1117: 80 (6.68%)	0.0381	<0.0001
Male	444: 3 (0.67%)	111: 3 (2.63%)	686: 36 (4.99%)	0.1018	<0.0001
Female	337: 3 (0.88%)	121: 3 (2.42%)	431: 44 (9.26%)	0.1964	<0.0001
Caucasian	662: 5 (0.75%)	205: 6 (2.84%)	985: 73 (6.90%)	0.0277	<0.0001
African American	66: 1 (1.49%)	16: 0 (0%)	63: 6 (8.70%)	>0.9999	0.0470

**Figure 2:**
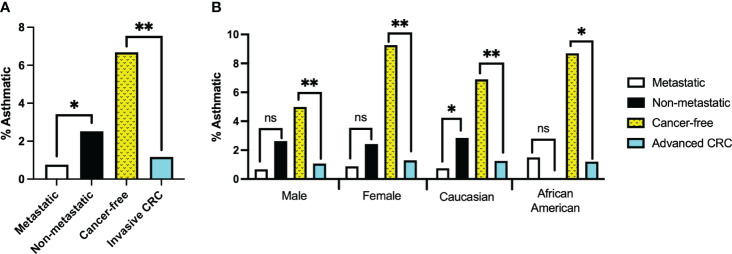
**(A)** Percentage of records with confirmed asthma diagnosis at least one year prior to colorectal cancer (CRC) diagnosis or prior to age 60 for Cancer-free group. Invasive CRC group represents combined records of the Metastatic and Non-metastatic cohorts. **(B)** Percentage of records with confirmed asthma diagnosis separated by sex or ethnicity. *, ** denotes p-values of <0.05 or <0.0001, respectively; ‘ns’ indicates no significant difference.

The framing of our search parameters allowed us to combine the two CRC cohorts into a single invasive CRC group of 1025 records which we compared against 1197 cancer-free patients. The average age at which patients received their initial CRC diagnosis was 60 years old. Using this as the upper age limit for asthma diagnosis in the cancer-free cohort, 80 patients were identified as asthmatic for the purposes of this study. Significantly fewer patients with invasive CRC disease had received a prior asthma diagnosis than in the cancer-free population. (P<0.0001, **Figure 2**).

Our tests revealed no clear driver for these results when stratifying for sex or race. Though males made up a larger percentage of the cancer-free group than either of the CRC patient cohorts, neither males nor females alone demonstrated a significant correlation between CRC metastasis and asthma diagnosis (P=0.1018 and 0.1964, respectively). However, they did reflect our findings that significantly fewer invasive CRC patients had prior asthma diagnoses (P<0.0001 in both cases).

Caucasians and African Americans comprised the majority of the patient cohort when stratifying for race. Most patient records in VUMC’s Synthetic Derivative are identified as Caucasian; even so, African Americans were considerably underrepresented in these cohorts compared to the patient population records in the Synthetic Derivative. As such, reliable statistical analysis could not be performed on African American records in isolation. It is no surprise then that analysis of our Caucasian patient records strongly reflected the findings of our unstratified cohorts (P=0.0277 (metastatic risk) and P<0.0001 (CRC Risk)).

## 4 Discussion

Our results found that patients previously diagnosed with asthma were significantly less likely to develop invasive CRC and had a reduced chance that their disease would progress to metastasis. While contrary to our initial hypothesis, it is not entirely unexpected. An inverse association between asthma and cancer has been reported for both pediatric acute lymphoblastic leukemia (ALL) (25) and for glioma (26). Moreover, a population-based study that looked at associations between having both hay fever and asthma with colorectal cancer mortality in 3 different cohorts suggested a modest decrease in mortality risk but suggested additional research was needed (27). The ALL study was a comprehensive one exploring links between ALL and multiple IgE-related conditions including rhinitis & sinusitis, food allergy and asthma. Additionally, the authors were able to report on genotypes for a range of SNPs of *IL4*, *IL13* and *IL4R* genes. Within ALL patients, the *IL4R* SNP rs1801275 was significantly associated with decreased asthma. This is particularly interesting because rs1801275 is a coding region SNP that results in a Q576R amino acid substitution in *IL4R*, which has been reported to lead to higher receptor activity for the arginine variant (20, 28). Indeed, there are multiple reports of patients having an increased likelihood of developing asthma and/or worse lung function if they carry the Q576R allele (21, 29–31). Interestingly, this arginine variant of IL4R (Q576R allele) was also associated with significantly increased risk of colorectal cancer in a British study (23), but there was no apparent association with mortality. Oddly, the authors of that paper considered the Q576R variant of IL4R has having reduced signaling capability, which is not the consensus amongst other studies.

One aspect that our study was not powered to address was the potential impact of asthma medication. Patients with chronic asthma can be treated with a variety of agents including corticosteroids, anti-histamines and bronchodilators. There is evidence for tumor-inhibiting roles for these agents in different settings. For example, studies have previously suggested that inhaled corticosteroids, a common preventative medication for asthma attacks, have a protective effect against lung cancer (17, 32–35). The glucocorticoid receptor is the primary target for synthesized corticosteroids and it is unclear if activation of the glucocorticoid receptor is linked to increased or reduced metastatic potential in colorectal cancer (36–40). Additionally, mast cells, which are one of the primary targets of anti-histamines, have also been considered as pro-tumorigenic in mouse models of colon cancer (41, 42). Since our study only covered patients that had been diagnosed before 2015, newer asthma medications that directly target the IL4/IL13/IL4R pathway such as dupilimab are not involved. However, it is worth noting that these agents are also shown to reduce cancer progression in mouse models (43) and it will be interesting to track patients who take these agents in the future.

Another possible explanation for our findings relates to the increased immune surveillance that could be active in patients with higher levels of IgE (16). There is some evidence that patients deficient in IgE have higher malignancy rates (44) however the corollary, that higher IgE levels are associated with lower risk, is not totally clear. In one large scale study, there was an apparently reduced risk for only chronic lymphocytic leukemia while higher or lower risks were not robustly shown for any other malignancy (45). In colon cancer, it has been known for some time that outcomes are affected by the levels of immune infiltrates within primary tumors (46), and this has resulted in an ‘immunoscore’ as a prognostic test (47).

Retrospective studies of deidentified records put themselves at risk of bias and inaccuracy by pulling from data taken for a purpose other than research, and often under circumstances where the patient and/or practitioner are stressed. It is therefore necessary to adopt rigorous protocols to ensure accuracy of examined records prior to addressing a study’s hypothesis. We attempted to collect similar data on patients diagnosed with other atopic diseases such as eczema, but were unable to acquire a sufficient sample size for analysis. This is unfortunate given the differences between atopic and non-atopic asthma for cancer risk reported by Woo et al. (17). Those results were seen in two independent Korean cohorts, and it would be interesting to know if this difference is also detectable in a Western population cohort. Similarly, our study would benefit from randomized selection but for our limited sample size. We noted that significantly fewer African Americans were represented in our study than expected. At the time of writing, approximately 9% of patients in the SD are identified as African American, and this percentage holds when examining all CRC patients in the database despite non-Hispanic Black people having a marked increase in CRC incidence nationwide (1). Reduced representation in our invasive CRC cohorts, and especially in our cancer-free group, may be explained by the longstanding socioeconomic inequities of VUMC’s serviced region, the effects of which go beyond the scope of this present study. Similar registries with equal access to cancer pathology data and suitable control population data are limited to the scope of a single healthcare provider. The widespread utilization of similar databases in the United States is stymied by necessary protections to patients’ privacy and limited technology to remove identifying data from the studied medical records (48). These investigative avenues will become more feasible as the Synthetic Derivative is expanded and similar resources are developed worldwide.

## Data availability statement

The data analyzed in this study is subject to the following licenses/restrictions: The primary data used for these analyses is maintained in VUMC’s Synthetic Derivative database, which can be accessed upon application to Vanderbilt Institute for Clinical and Translational Research. Requests to access these datasets should be directed to research.support.services@vumc.org.

## Ethics statement

The studies involving humans were approved by Vanderbilt University Medical Center IRB. The studies were conducted in accordance with the local legislation and institutional requirements. Written informed consent for participation was not required from the participants or the participants’ legal guardians/next of kin in accordance with the national legislation and institutional requirements.

## Author contributions

JB: Data curation, Formal Analysis, Investigation, Methodology, Writing – original draft. KM: Investigation, Writing – review & editing. KW: Investigation, Writing – review & editing. BF: Conceptualization, Funding acquisition, Supervision, Writing – review & editing.
