# Supplementary material for: Asthma is associated with a lower incidence of metastatic colorectal cancer in a US patient cohort
Source: Front Oncol. 2023 Oct 4;13:1253660. doi: 10.3389/fonc.2023.1253660 (PMC10584144; doi:10.3389/fonc.2023.1253660)
Supplement: Supplementary file 1 [file Table_1.docx]

Supplementary Material

Asthma is associated with a lower incidence of metastatic colorectal cancer in a US patient cohort

**Jacob Beckstead, Kunaal Mehrotra, Kayla Wilson, and Barbara Fingleton***

Program in Cancer Biology, Department of Pharmacology, Vanderbilt University, Nashville, Tennessee, USA

*** Corresponding Author:** [Barbara.fingleton@vanderbilt.edu](mailto:Barbara.fingleton@vanderbilt.edu)

**Supplementary Table 1.** ICD 9 and 10 codes used as inclusion criteria for colorectal cancer cohorts. Instances of these codes must occur between January 1^st^, 2005 and December 31^st^, 2015 to be included.

| **ICD 9** |
| --- |
| 153-Malignant neoplasm of colon  153.0-Malignant neoplasm of hepatic flexure  153.1-Malignant neoplasm of transverse colon  153.2-Malignant neoplasm of descending colon  153.3-Malignant neoplasm of sigmoid colon  153.4-Malignant neoplasm of cecum  153.6-Malignant neoplasm of ascending colon  153.7-Malignant neoplasm of splenic flexure  153.8-Malignant neoplasm of other specified sites of large intestine  153.9-Malignant neoplasm of colon, unspecified site  154-Malignant neoplasm of rectum, rectosigmoid junction, and anus  154.0-Malignant neoplasm of rectosigmoid junction  154.8-Malignant neoplasm of other sites of rectum, rectosigmoid junction, and anus |
| **ICD 10** |
| C18.0-Malignant neoplasm of cecum  C18.2-Malignant neoplasm of ascending colon  C18.3-Malignant neoplasm of hepatic flexure  C18.4-Malignant neoplasm of transverse colon  C18.5-Malignant neoplasm of splenic flexure  C18.6-Malignant neoplasm of descending colon  C18.7-Malignant neoplasm of sigmoid colon  C18.8-Malignant neoplasm of overlapping sites of colon  C18.9-Malignant neoplasm of colon, unspecified  C19-Malignant neoplasm of rectosigmoid junction  C21.8-Malignant neoplasm of overlapping sites of rectum, anus and anal canal |

**Supplementary Table 2.** ICD 9 and 10 codes and relevant keywords serving as inclusive criteria for the metastatic colorectal cancer cohort. To qualify for inclusion, records must have at least two instances of these criteria.

| **ICD 9** |
| --- |
| 196-Secondary and unspecified malignant neoplasm of lymph nodes  196.0-Secondary and unspecified malignant neoplasm of lymph nodes of head, face, and neck  196.1-Secondary and unspecified malignant neoplasm of intrathoracic lymph nodes  196.2-Secondary and unspecified malignant neoplasm of intra-abdominal lymph nodes  196.3-Secondary and unspecified malignant neoplasm of lymph nodes of axilla and upper limb  196.5-Secondary and unspecified malignant neoplasm of lymph nodes of inguinal region and lower limb  196.6-Secondary and unspecified malignant neoplasm of intrapelvic lymph nodes  196.8-Secondary and unspecified malignant neoplasm of lymph nodes of multiple sites  196.9-Secondary and unspecified malignant neoplasm of lymph nodes, site unspecified  197-Secondary malignant neoplasm of respiratory and digestive systems  197.0-Secondary malignant neoplasm of lung  197.1-Secondary malignant neoplasm of mediastinum  197.2-Secondary malignant neoplasm of pleura  197.3-Secondary malignant neoplasm of other respiratory organs  197.4-Secondary malignant neoplasm of small intestine including duodenum  197.5-Secondary malignant neoplasm of large intestine and rectum  197.6-Secondary malignant neoplasm of retroperitoneum and peritoneum  197.7-Malignant neoplasm of liver, secondary  197.8-Secondary malignant neoplasm of other digestive organs and spleen  198-Secondary malignant neoplasm of other specified sites  198.0-Secondary malignant neoplasm of kidney  198.1-Secondary malignant neoplasm of other urinary organs  198.2-Secondary malignant neoplasm of skin  198.3-Secondary malignant neoplasm of brain and spinal cord  198.4-Secondary malignant neoplasm of other parts of nervous system  198.5-Secondary malignant neoplasm of bone and bone marrow  198.6-Secondary malignant neoplasm of ovary  198.7-Secondary malignant neoplasm of adrenal gland  198.8-Secondary malignant neoplasm of other specified sites  198.81-Secondary malignant neoplasm of breast  198.82-Secondary malignant neoplasm of genital organs  198.89-Secondary malignant neoplasm of other specified sites |
| **ICD 10** |
| C77.0-Secondary and unspecified malignant neoplasm of lymph nodes of head, face and neck  C77.1-Secondary and unspecified malignant neoplasm of intrathoracic lymph nodes  C77.2-Secondary and unspecified malignant neoplasm of intra-abdominal lymph nodes  C77.3-Secondary and unspecified malignant neoplasm of axilla and upper limb lymph nodes  C77.4-Secondary and unspecified malignant neoplasm of inguinal and lower limb lymph nodes  C77.5-Secondary and unspecified malignant neoplasm of intrapelvic lymph nodes  C77.8-Secondary and unspecified malignant neoplasm of lymph nodes of multiple regions  C77.9-Secondary and unspecified malignant neoplasm of lymph node, unspecified  C78.00-Secondary malignant neoplasm of unspecified lung  C78.1-Secondary malignant neoplasm of mediastinum  C78.2-Secondary malignant neoplasm of pleura  C78.39-Secondary malignant neoplasm of other respiratory organs  C78.4-Secondary malignant neoplasm of small intestine  C78.5-Secondary malignant neoplasm of large intestine and rectum  C78.6-Secondary malignant neoplasm of retroperitoneum and peritoneum  C78.7-Secondary malignant neoplasm of liver and intrahepatic bile duct  C78.7-Secondary malignant neoplasm of liver and intrahepatic bile duct  C78.89-Secondary malignant neoplasm of other digestive organs  C79.00-Secondary malignant neoplasm of unspecified kidney and renal pelvis  C79.11-Secondary malignant neoplasm of bladder  C79.19-Secondary malignant neoplasm of other urinary organs  C79.2-Secondary malignant neoplasm of skin  C79.31-Secondary malignant neoplasm of brain  C79.32-Secondary malignant neoplasm of cerebral meninges  C79.49-Secondary malignant neoplasm of other parts of nervous system  C79.51-Secondary malignant neoplasm of bone  C79.52-Secondary malignant neoplasm of bone marrow  C79.60-Secondary malignant neoplasm of unspecified ovary  C79.70-Secondary malignant neoplasm of unspecified adrenal gland  C79.81-Secondary malignant neoplasm of breast  C79.82-Secondary malignant neoplasm of genital organs  C79.89-Secondary malignant neoplasm of other specified sites |
| **Keywords in Pathology Reports** |
| pN1  pN2 |

**Supplemental Table 3.** ICD 9 and 10 codes and keywords used as exclusion criteria for the non-metastatic invasive CRC cohort.

| **ICD 9** | | |
| --- | --- | --- |
| 196-Secondary and unspecified malignant neoplasm of lymph nodes  196.0-Secondary and unspecified malignant neoplasm of lymph nodes of head, face, and neck  196.1-Secondary and unspecified malignant neoplasm of intrathoracic lymph nodes  196.2-Secondary and unspecified malignant neoplasm of intra-abdominal lymph nodes  196.3-Secondary and unspecified malignant neoplasm of lymph nodes of axilla and upper limb  196.5-Secondary and unspecified malignant neoplasm of lymph nodes of inguinal region and lower limb  196.6-Secondary and unspecified malignant neoplasm of intrapelvic lymph nodes  196.8-Secondary and unspecified malignant neoplasm of lymph nodes of multiple sites  196.9-Secondary and unspecified malignant neoplasm of lymph nodes, site unspecified  197-Secondary malignant neoplasm of respiratory and digestive systems  197.0-Secondary malignant neoplasm of lung  197.1-Secondary malignant neoplasm of mediastinum  197.2-Secondary malignant neoplasm of pleura  197.3-Secondary malignant neoplasm of other respiratory organs  197.4-Secondary malignant neoplasm of small intestine including duodenum  197.5-Secondary malignant neoplasm of large intestine and rectum  197.6-Secondary malignant neoplasm of retroperitoneum and peritoneum  197.7-Malignant neoplasm of liver, secondary  197.8-Secondary malignant neoplasm of other digestive organs and spleen  198-Secondary malignant neoplasm of other specified sites  198.0-Secondary malignant neoplasm of kidney  198.1-Secondary malignant neoplasm of other urinary organs  198.2-Secondary malignant neoplasm of skin  198.3-Secondary malignant neoplasm of brain and spinal cord  198.4-Secondary malignant neoplasm of other parts of nervous system  198.5-Secondary malignant neoplasm of bone and bone marrow  198.6-Secondary malignant neoplasm of ovary  198.7-Secondary malignant neoplasm of adrenal gland  198.8-Secondary malignant neoplasm of other specified sites  198.81-Secondary malignant neoplasm of breast  198.82-Secondary malignant neoplasm of genital organs  198.89-Secondary malignant neoplasm of other specified sites | | |
| **ICD 10** | | |
| C77.0-Secondary and unspecified malignant neoplasm of lymph nodes of head, face and neck  C77.1-Secondary and unspecified malignant neoplasm of intrathoracic lymph nodes  C77.2-Secondary and unspecified malignant neoplasm of intra-abdominal lymph nodes  C77.3-Secondary and unspecified malignant neoplasm of axilla and upper limb lymph nodes  C77.4-Secondary and unspecified malignant neoplasm of inguinal and lower limb lymph nodes  C77.5-Secondary and unspecified malignant neoplasm of intrapelvic lymph nodes  C77.8-Secondary and unspecified malignant neoplasm of lymph nodes of multiple regions  C77.9-Secondary and unspecified malignant neoplasm of lymph node, unspecified  C78.00-Secondary malignant neoplasm of unspecified lung  C78.1-Secondary malignant neoplasm of mediastinum  C78.2-Secondary malignant neoplasm of pleura  C78.39-Secondary malignant neoplasm of other respiratory organs  C78.4-Secondary malignant neoplasm of small intestine  C78.5-Secondary malignant neoplasm of large intestine and rectum  C78.6-Secondary malignant neoplasm of retroperitoneum and peritoneum  C78.7-Secondary malignant neoplasm of liver and intrahepatic bile duct  C78.7-Secondary malignant neoplasm of liver and intrahepatic bile duct  C78.89-Secondary malignant neoplasm of other digestive organs  C79.00-Secondary malignant neoplasm of unspecified kidney and renal pelvis  C79.11-Secondary malignant neoplasm of bladder  C79.19-Secondary malignant neoplasm of other urinary organs  C79.2-Secondary malignant neoplasm of skin  C79.31-Secondary malignant neoplasm of brain  C79.32-Secondary malignant neoplasm of cerebral meninges  C79.49-Secondary malignant neoplasm of other parts of nervous system  C79.51-Secondary malignant neoplasm of bone  C79.52-Secondary malignant neoplasm of bone marrow  C79.60-Secondary malignant neoplasm of unspecified ovary  C79.70-Secondary malignant neoplasm of unspecified adrenal gland  C79.81-Secondary malignant neoplasm of breast  C79.82-Secondary malignant neoplasm of genital organs C79.89-Secondary malignant neoplasm of other specified sites | | |
| **Keywords** | | |
| well differentiated neuroendocrine  stage III rectal  stage III colorectal  stage III colon  0n1  0n2  0n3  1n1  1n2 | 1n3  2n1  2n2  2n3  3n1  3n2  3n3  N1  N2 | pN1  pN2  Stage IV rectal  Stage IV colon  Stage IV colorectal  T3 N1 rectal  T3N1 rectal  uN1 rectal |
